# Soil conditions on bacterial wilt disease affect bacterial and fungal assemblage in the rhizosphere

**DOI:** 10.1186/s13568-022-01455-1

**Published:** 2022-08-29

**Authors:** Xiaojiao Liu, Liehua Liu, Jie Gong, Lixin Zhang, Qipeng Jiang, Kuo Huang, Wei Ding

**Affiliations:** 1 State Key Laboratory of Silkworm Genome Biology, Key Laboratory of Sericultural Biology and Genetic Breeding in Ministry of Agriculture, College of Sericulture, Textile and Biomass Science, Southwest University, Chongqing, 400715 China; 2 Microecological Process and Regulation Key Laboratory, College of Plant Protection, Southwest University, Chongqing, 400715 China; 3 Zhengzhou Tobacco Research Institute of China National Tobacco Corporation, Henan, 450001 China

**Keywords:** Microbial assemblage, Soil condition, Rhizosphere, Bacterial wilt disease, Illumina sequencing

## Abstract

Natural soil has the ability to suppress the soil-borne pathogen to a certain extent, and the assemblage of soil microbiome plays a crucial role in maintaining such ability. Long-term monoculture accelerates the forms of soil microbiome and leads to either disease conducive or suppressive soils. Here, we explored the impact of soil conditions on bacterial wilt disease (healthy or diseased) under long-term tobacco monoculture on the assemblage of bacterial and fungal communities in bulk and rhizosphere soils during the growth periods. With Illumina sequencing, we compared the bacterial and fungal composition of soil samples from tobacco bacterial wilt diseased fields and healthy fields in three growth periods. We found that *Proteobacteria* and *Ascomycota* were the most abundant phylum for bacteria and fungi, respectively. Factors of soil conditions and tobacco growth periods can significantly influence the microbial composition in bulk soil samples, while the factor of soil conditions mainly determined the microbial composition in rhizosphere soil samples. Next, rhizosphere samples were further analyzed with LEfSe to determine the discriminative taxa affected by the factor of soil conditions. For bacteria, the genus *Ralstonia* was found in the diseased soils, whereas the genus *Flavobacterium* was the only shared taxon in healthy soils; for fungi, the genus *Chaetomium* was the most significant taxon in healthy soils. Besides, network analysis confirmed that the topologies of networks of healthy soils were higher than that of diseased soils. Together, our results suggest that microbial assemblage in the rhizosphere will be largely affected by soil conditions especially after long-term monoculture.

**Supplementary Information:**

The online version contains supplementary material available at 10.1186/s13568-022-01455-1.

## Introduction

Bacterial wilt, caused by *Ralstonia solanacearum*, is one typical soil-borne disease and can bring severe losses to agricultural crops (Genin and Denny 2012; Jiang et al. 2017). In addition, long-term monoculture is more likely to cause rapid accumulation of *R. solanacearum* in soil (Chen et al. 2020a). Until now, there is currently no effective chemical pesticide to manage this disease (Liu et al. 2013). As an attractive alternative, antagonistic microbes were introduced as potential biocontrol agents (Guo et al. 2014). A number of antagonism studies have dealt with the pairwise interactions between beneficial and pathogenic microbes (reviewed by de Boer 2017). Unfortunately, in real communities, the complexity of the soil environment may largely decrease their inefficiency of pathogen suppression (Mallon et al. 2015; de Boer 2017). However, the phenomenon of ‘disease-suppressive soils’ shows an ideal model by which plant protection can be triggered by soil microbes (reviewed by Wang and Li 2019a). Hence, a better knowledge of understanding the positive functions from indigenous microbial communities in the soil is essential for a sustainable and effective bacterial wilt management strategy.

Indigenous microbial communities in the soil forms complex networks and manipulates plant health (Berendsen et al. 2012). Based on the composition of the resident microbial community, a biological barrier is formed, with microbes interacting with pathogens and defending against invasion by pathogens near the root surface (Raaijmakers et al. 2009; Fu et al. 2017). Therefore, assessing the relationship between the soil community and crop morbidity is a critical step toward understanding potential impacts of these communities on plant health (Rosenzweig et al. 2012; Cha et al. 2016; Xiao et al. 2018). It has been revealed that species-rich biomes are more resistant than species-poor biomes to pathogen invasions (Wei et al. 2015) and high incidence of soil-borne diseases could be due to the deterioration of the soil microecological environment (Gao et al. 2020). Additionally, tobacco farmlands with high biodiversity were more resistant to pathogen infection (Wang et al. 2019).

Soil microbial community changes dramatically during plant growth (Lundberg et al. 2012; Xiong et al. 2015). It is important to understand the composition and interaction of microbial communities during plant development (Chaparro et al. 2014). Evidence suggests that *Arabidopsis* at different developmental stages can culture specific rhizosphere microbiome members (Yuan et al. 2015). Similarly, the rhizosphere microbiome characteristics of maize change with growth stage (Li et al. 2014). During infection by bacterial wilt, the composition of the microbial communities in the rhizosphere of tomato at different growth stages is significantly different (Wei et al. 2018). Research has demonstrated that plant is a unique determinate of community structure in the rhizosphere at early stages, but that these differences in the microbiome disappear as plant develops (Inceoğlu et al. 2011).

Here we report the results of the bacterial wilt diseased and healthy soil microbial assemblages at different growth stages of tobacco. We included the soils collected from diseased fields, and the ones from healthy fields. Bulk soils were collected in March and rhizosphere soils were collected in July and September. We examined the soil biochemical properties and microbial compositions. We investigated the influences of soil conditions and tobacco growth periods on the bacterial and fungal assemblage.

## Materials and methods

### Site description and sampling

The sampling sites were located in Chongqing, China. Detailed geographical information was listed in Additional file 1: Table S1. Soils were loamy and had a history of continuous tobacco cropping over 20 years. The variety of tobacco was Yunyan 87, which is a hybrid of Yunyan 2 (origin: China) and K326 (origin: USA) (Chen et al. 2020b). Fertilizers and pesticides were applied under the same standards established by the Chongqing Tobacco Corporation. Bacterial wilt diseases caused by *R. solanacearum* severely affected the production in sites of Wuling area in recent years, whereas there was no soil-borne disease in sites of Qinba area. In this study, soils from Wuling area were treated as bacterial-wilt diseased soils while soils from Qinba area were healthy soils.

Soil samples were collected in March, July and September 2017, which represented the period before transplanting tobacco seedling into the soil, the start of budding stage and during mature stage of tobacco, respectively. As there was no tobacco planting in March, bulk soil samples were collected at a depth of 10–20 cm; besides, rhizosphere soil samples were collected in July and September following the methods mentioned in our previous study (Liu et al. 2016). Soil samples were sieved (2 mm) and stored at − 80 °C for DNA extraction within one week. Additionally, in each March sample, one subsample was previously prepared for analysis of soil physical and chemical properties.

### Soil physical and chemical properties analyses

All soil physical–chemical properties were determined according to Bao (2010). Briefly, soil pH was measured with a glass electrode in deionized water suspensions (soil: water = 1:2.5, w/v). The soil organic matter (SOM) was assayed by the potassium dichromate method. Total nitrogen (TN) was estimated by the semi-micro Kjeldahl method. Available nitrogen (AvN), phosphorus (AvP) and potassium (AvK) were determined using the NaOH hydrolyzation diffusion method, the molybdenum blue colorimetric method and the flame photometer method, respectively. Exchangeable calcium (ExCa) and magnesium (ExMg) were measured by the inductively coupled plasma atomic emission spectrometer (ICP-AES) method.

### Tobacco bacterial wilt disease investigation

Bacterial wilt symptoms of tobacco were apparently observed in the diseased fields (Wuling area) from June to October. In September of both 2016 and 2017, the end stage of bacterial wilt outbreak, around 40 plants in each sampling site were evaluated to calculate the disease incidence according to (Qi et al. 2020). Meanwhile, due to the healthy state of tobacco in the healthy fields (Qinba area), we only described the disease incidence of diseased fields.

### DNA extraction and sequencing processing

Soil microbial DNA was extracted from 0.5 g of soil using a FastDNA Spin kit (MP Biomedicals, Santa Ana, CA, USA) following the manufacturer’s protocol. DNA quality and concentration were evaluated by Nanodrop (NanoDrop ND-1000, Thermo Fisher, Wilmington, DE, USA). Amplification of the V4-region of the bacterial 16S rRNA gene was performed using primers 515F and 806R (Bergmann et al. 2011). Fungal ITS1 region was amplified using primers ITS1F and ITS2R (Bellemain et al. 2010). The PCR reaction was carried out in triplicate Each sample was amplified under the following conditions: 94 °C for 5 min, followed by 35 cycles including 94 °C for 45 s, 50 °C for 60 s and 72 °C for 90 s, then 72 °C for 10 min for bacteria; and 94 °C for 3 min, followed by 30 cycles including 94 °C for 30 s, 55 °C for 30 s, and 72 °C for 30 s, then 72 °C for 10 min for fungi. The PCR products were mixed and purified by Agarose Gel DNA purification kit (TaKaRa, Dalian, China). The amplicons were equally combined to produce two separate PCR pools (keeping bacterial and fungal amplicons separate) that were sent to Majorbio Bio-pharm Technology Co., Ltd. (Shanghai, China) for 300-bp paired-end sequencing on an Illumina MiSeq platform in two separate runs. The raw reads were deposited into the NCBI short-reads archive database under accession number SRP270966.

### Bioinformatics analysis

The Raw Illumina FASTQ files were demultiplexed, quality filtered, and analyzed using QIIME v1.7.0 (Quantitative Insights Into Microbial Ecology) (Caporaso et al. 2010). Operational taxonomic units (OTUs) were grouped with a threshold of 97% pairwise identity by QIIME. Any sample that had fewer than 20 useable reads was discarded, resulting the unnormalized usable OTU table. Based on this table, a rarefied table was made by rarefying it to the minimum reads. Next, a frequency table was created normalized by transforming the data into relative abundance (Total Sum Scaling normalization (Lundberg et al. 2012)).

Microbial α-diversity and β-diversity analysis were performed using the free online platform of Majorbio Cloud Platform (www.majorbio.com). Indies of Sobs, Shannon, Ace and Chao1 were calculated as microbial α-diversity. The dissimilarity of the microbial communities was determined using principal coordinate analysis (PCoA) on Bray–Curtis distance. Permutational multivariate analyses of variance (PERMANOVA) using Bray–Curtis distances with 999 permutations was performed within each sample type to explore the percentage of variance explained by the factors of soil conditions and tobacco growth periods.

The LEfSe (Linear Discriminate Analysis (LDA) Effect Size) (Segata et al. 2011) algorithm was performed on the website (http://huttenhower.sph.harvard.edu/galaxy). A factorial Kruskal–Wallis sum-rank test (α = 0.05) was used to identify taxa with significant differential abundances between categories (using all-against-all comparisons) for the sequencing data collected in both July and September. The value of LDA was used to estimate the effect size of each differentially abundant feature. Taxa with logarithmic LDA value over 4.00 were selected to perform histogram figures. The number and relative abundance of shared taxa in healthy soils were further shown in Venn diagram and histogram.

Network analyses were performed using the molecular ecological network analyses (MENA) pipeline (http://ieg4.rccc.ou.edu/mena) with default settings and recommended similarity thresholds (Deng et al. 2012). The co-occurrence networks were visualized in Gephi (Ver. 0.9.2) (Bastian et al. 2009). OTUs with relative abundance over 0.1% in rhizosphere samples of July and September were used for network construction.

### Statistical analysis

All statistical analyses were performed in SPSS Statistics 17.0 software program (SPSS Inc., USA). Significant differences in physical and chemical properties of soil samples and alpha diversity indices of soil microbial community across sampling region were determined by independent-sample t test.

## Results

### Bulk soil properties and tobacco bacterial wilt disease incidence

Differences of physical and chemical properties of bulk soil collected in March between diseased and healthy soils were shown in Table 1. Interestingly, although there were many differences in the chemical properties (AvN, AvK etc.), none of them reached the significance (*p* > 0.05).

**Table 1:** Physical and chemical properties of soil samples (mean ± SE, N = 9)

Fields	pH	SOM (g kg^−1^)	TN (g kg^−1^)	AvN (mg kg^−1^)	AvP (mg kg^−1^)	AvK (mg kg^−1^)	ExCa (g kg^−1^)	ExMg (g kg^−1^)
Healthy	5.52 ± 0.08	22.65 ± 2.36	1.50 ± 0.11	105.84 ± 9.43	40.33 ± 5.17	387.78 ± 52.09	1.65 ± 0.18	0.20 ± 0.03
Diseased	5.72 ± 0.17	22.42 ± 0.89	1.54 ± 0.07	123.11 ± 6.13	39.37 ± 7.60	495.00 ± 62.26	1.60 ± 0.16	0.17 ± 0.03

Disease incidences of tobacco bacterial wilt in diseased soils were recorded in July of both 2016 and 2017. There were over 50% of tobacco plants showing typical bacterial-wilt symptoms in all the sampling sites (Table 2).

**Table 2:** Two-year tobacco bacterial wilt disease incidence of collection fields (mean ± SE, N = 3)

Fields	Disease incidence (%) in 2016	Disease incidence (%) in 2017
Healthy	–	–
Diseased	64.26 ± 3.12	62.29 ± 11.56

### Soil microbial community α-diversity

For bacteria, a total of 27,609 raw sequence reads was obtained through 16S rRNA gene sequencing. There was no significant difference in the richness or evenness indices of the bacterial community in both March and September samples (Additional file 1: Table S2). The Shannon index was significantly higher in the healthy soils than diseased soils in July (*p* < 0.05).

For fungi, a total of 30,581 raw sequence reads was obtained through ITS sequencing. All the diversity indices of the fungal community in the diseased soils gradually decreased throughout the plant growth periods, and was lowest by September (Additional file 1: Table S2). In contrast, the richness and diversity indices of the healthy samples gradually increased, reaching the highest value in July.

### Soil microbial community β-diversity

**Fig. 1:**
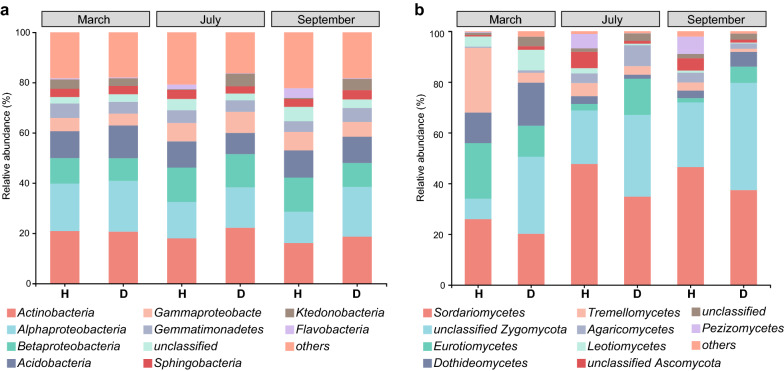
Relative abundance of the top-10 a) bacterial and b) fungal classes for samples collected form healthy (H) and diseased (D) fields in March, July and September

*Proteobacteria* (36.94 ± 0.64%; mean ± SE) was the most dominated bacterial phylum in soil samples, followed by *Actinobacteria* (19.42 ± 0.92%). Relative abundance of bacterial classes changed during different tobacco growth stages in both healthy and diseased soils (Fig. 1a). For healthy soils, a clear shift was seen from dominance of *Alphaproteobacteria* (18.89%) in the bulk soil samples collected in March to that of *Betaproteobacteria* (13.65%) in the rhizosphere samples collected in both July and September. For diseased soils, the decrease in relative abundance of *Alphaproteobacteria* (17.94%) were also seen in rhizosphere samples; however, this compensation of *Betaproteobacteria* (13.21%) was only in July samples.

*Ascomycota* (60.35 ± 2.81%) was the most abundant fungal phylum in soil samples, including *Sordariomycetes* (35.42 ± 4.47%), *Eurotiomycetes* (9.85 ± 3.17%), *Dothideomycetes* (7.09 ± 2.50%), *Leotiomycetes* (2.66 ± 1.22%) and *Pezizomycetes* (2.16 ± 1.31%) listed as top-10 classes (Fig. 1b). *Sordariomycetes* were higher in healthy soils (47.08 ± 0.50%) than in diseased ones (36.12 ± 1.06%). For the rhizosphere samples, there was a consistent enrichment of *Eurotiomycetes* in diseased soils (10.37 ± 3.92%), while *Pezizomycetes* was largely seen in healthy soils (6.27 ± 0.58%).

**Fig. 2:**
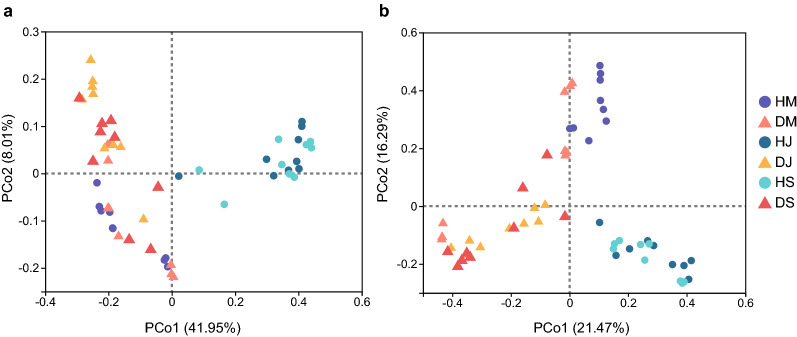
PCoA of the soil **a** bacterial and **b** fungal community compositions. HM/DM: soils collected from healthy (H) and diseased (D) fields in March; HJ/DJ: soils collected from healthy (H) and diseased (D) fields in July; HS/DS: soils collected from healthy (H) and diseased (D) fields in September

Besides, effects of soil conditions on both bacterial and fungal composition were clearly seen from the PCoA analysis (Fig. 2 and Additional file 1: Table S1). PERMANOVA analyses were further confirmed with significance of the factor of soil conditions in samples with and without bulk soil samples (*p* < 0.001; Additional file 1: Tables S3 and S4). Specifically, PCoA explained 22% and 12% of total variation in compositions of bacterial and fungal bacterial communities in rhizosphere soils. Additionally, there were few effects of tobacco growth periods on both bacterial and fungal community compositions in rhizosphere samples (Additional file 1: Table S4).

### Rhizosphere microbial taxa discriminated by soil conditions

**Fig. 3:**
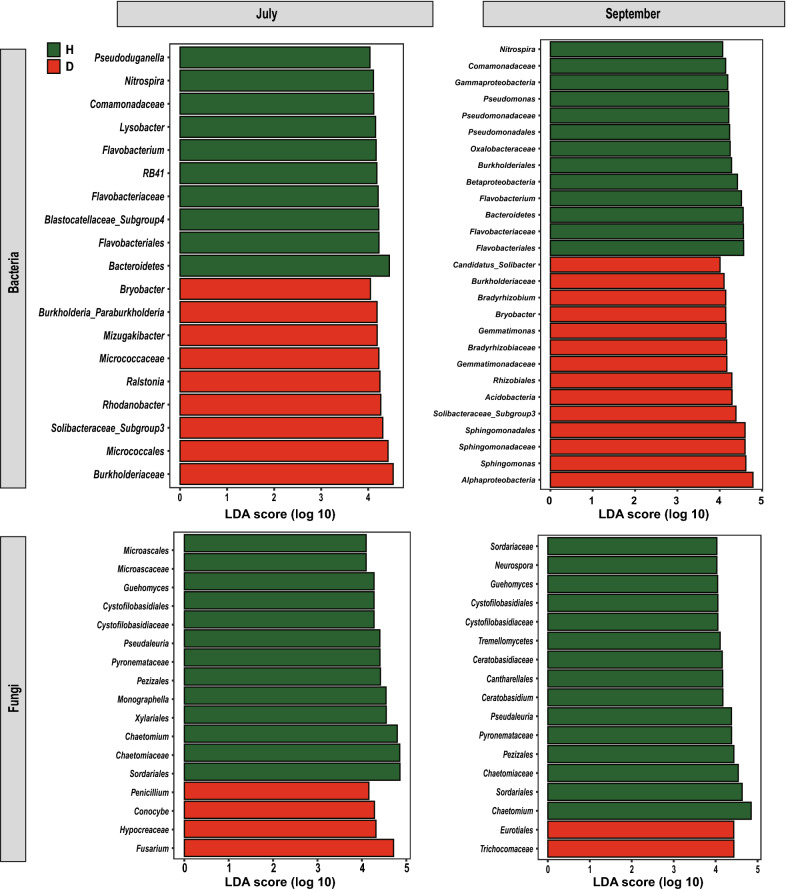
Histogram of discriminative bacterial and fungal taxa in healthy (H) and diseased (D) rhizosphere samples. Rhizosphere samples indicated the soil samples collected in July and September

**Fig. 4:**
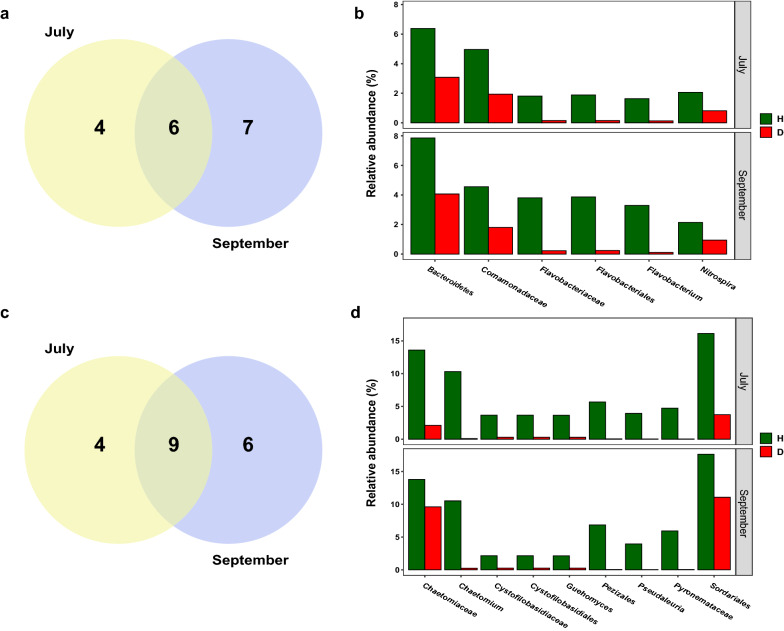
Venn diagram showing the number of discriminative bacterial (**a**) and fungal (**c**) taxa in healthy soils and histogram showing relative abundance of shared bacterial (**b**) and fungal (**d**) taxa in both healthy (H) and diseased (D) samples

Rhizosphere soil samples collected in July and September were further compared with LEfSe to determine the discriminative taxa affected by the factor of soil conditions. In July samples, there were 10 taxa found in the heathy soils and 9 in the diseased soils (Fig. 3). The genus *Ralstonia*, to which the pathogen *R. solanacearum* belongs, was found in the diseased soils (logarithmic LDA = 4.25, *p* = 0.02). The most significant taxon in the healthy soils was the phylum *Bacteroidetes* (logarithmic LDA = 4.45, *p* < 0.001). In September samples, a total of 35 taxa were found: 13 taxa in the healthy soils and 14 taxa in the diseased soils (Fig. 3). The class *Alphaproteobacteria* (logarithmic LDA = 4.78, *p* = 0.02) was the most significant taxon, followed by the genus *Sphingomonas* (logarithmic LDA = 4.62, *p* < 0.001) in diseased soils, while the order *Flavobacteriales* (logarithmic LDA = 4.78, *p* < 0.001) had the largest effects in healthy soils. Among those discriminative taxa in healthy soils, there were 6 taxa shown in both July and September samples (Fig. 4a), and *Flavobacterium* (in July samples, 1.64 ± 0.11%; in September samples, 3.29 ± 0.42%) was the only shared genera (Fig. 4b).

With respect to fungi, the LEfSe analysis revealed that there were more discriminative taxa in healthy soils than diseased soils. In July samples, 13 taxa were identified for the healthy soils, and 4 taxa for the diseased soils (Fig. 3). The genus *Fusarium* (logarithmic LDA = 4.72, *p* = 0.02) was found as the most significant taxon in diseased soils, while the order *Sordariales* (logarithmic LDA = 4.86, *p* < 0.001) had the largest effects in healthy soils. In September samples, there were 15 taxa found in the heathy soils and 2 in the diseased soils (Fig. 3). Among those discriminative taxa in healthy soils, there were 9 taxa shown in both July and September samples (Fig. 4c). The genus *Chaetomium* (in July samples, 10.32 ± 3.22%; in September samples, 10.52 ± 6.23%) was the most significant genus in healthy soils collected in both July and September (Fig. 4d).

### Rhizosphere microbial networks explained by soil conditions

**Fig. 5:**
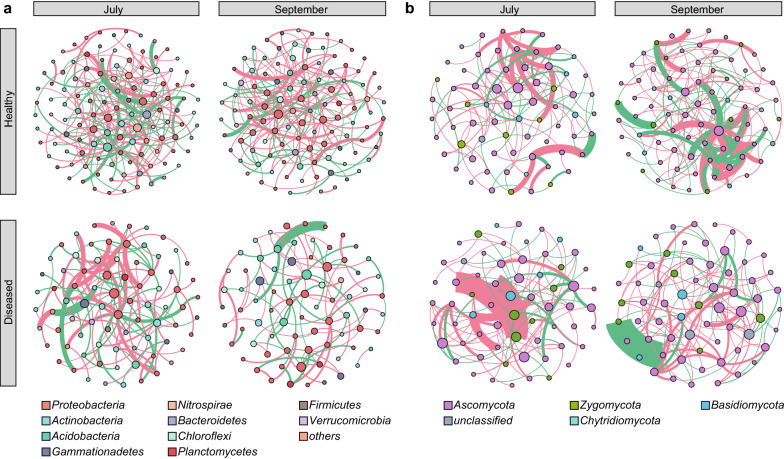
Co-occurrence patterns of** a** bacteria and **b** fungi in healthy and diseased rhizosphere samples. Nodes indicate taxonomic affiliation at phylum level. Red lines indicate positive correlations, and green lines indicate negative correlations. The size of each node is proportional to the betweenness centrality. Detailed properties of network indices were listed in Additional file 1: Table S5

For bacterial networks, both the network size and the degree of connectivity showed large differences between healthy and diseased soils (Fig. 5a). The topologies of the healthy networks, namely, the number of nodes and the number of links, were higher than that of the diseased networks (Additional file 1: Table S5). In total, there were 231 nodes and 433 links in healthy soils, whereas 156 nodes and 216 links in diseased soils (Fig. 5a). Moreover, using betweenness centrality, *Flavobacterium* (OTU3316, OTU4570) and *Pseudomonas* (OTU1200, OTU3398, OTU9417) were found as key genera in the networks of healthy soils.

For fungal networks, the number of nodes and links was lower in diseased soils than healthy soils (Fig. 5b and Additional file 1: Table S5). In July samples, there were 125 links and 70 nodes in healthy soils, and 115 links and 63 nodes in diseased soils (Fig. 5b). In September sample, the network of healthy soils contained 163 links and 81 nodes, while the network of diseased soils contained 128 links and 65 nodes (Fig. 5b). High betweenness centrality values were found for the genera *Fusarium* (OTU672, OTU4590) and *Mortierella* (OTU1037, OTU1987) in the networks of healthy soils.

## Discussion

Here, we investigated bacterial and fungal communities from the early growth stage to the last two growth periods and the microbial community in the later stages of tobacco growth plays an integral role in plant-pathogen interactions. Increasing evidence has shown that the rhizosphere microbial community plays an indispensable role in relieving nutrient stress and responding to pathogenic micro-invasion by using root exudates from plant roots (Okubo et al. 2016). Plants are able to recruit specific bacteria and fungi for defense against bacterial wilt in the rhizosphere (Lareen et al. 2016). Additionally, the specific selection of microbiome by plants in the rhizosphere mainly differs at different developmental stages (Yang and Crowley 2000; Bulgarelli et al. 2012). Infection by pathogenic bacteria is the main cause of plant recruitment of beneficial microorganisms in the rhizosphere (Bakker et al. 2013), and the antagonistic effect on pathogens is enhanced during plant development (Hu et al. 2020). Specific resident plant rhizosphere bacterial communities that adapt to plants play important roles in both optimize growth and protecting against infection by pathogens. The recruitment of beneficial microorganisms can also change the physiological function of plants to allow them to resist aerial pathogens (Kumar et al. 2012).

Although the rhizosphere effects on microbial assemblage is proved to be crucial in plant health, it also have been reported that the initial variation in soil bacterial composition and functioning can determine the outbreak of bacterial wilt disease (Wei et al. 2019). And thus, understanding the difference of microbial community in healthy and diseased soils are important regarding to plant-pathogen interactions. In this study, we confirmed significant shifts in the diversity and abundance of bacterial and fungal communities associated with healthy and diseased soils. Indeed, there are increasing studies focusing on the microbial indicators associated with the suppression of tobacco bacterial wilt (Liu et al. 2016; She et al. 2017). Here, we used LEfSe and co-occurrence network to investigate the keystone species as well.

Network analysis have been widely used to determine the association and co-occurrence complexity of microorganisms (Su et al. 2020). Our study shows that the rhizosphere soil co-occurance network of healthy tobacco plants is more complicated than that of diseased tobacco plants. This is consistent with previous research results (Yang et al. 2017). Indeed, microbial communities with relatively high diversity have better resistance to invasion by pathogenic bacteria (Hu et al. 2020). Interactions between microbial species can affect disease dynamics by changing the relative and absolute density of pathogens in the host-associated microbiome (Mendes et al. 2011; Mueller and Sachs 2015). In the healthy and diseased rhizosphere soil networks, we observed positive interactions between nodes, indicating niche overlap, as well as negative interactions, which suggest competition or exclusion (Faust and Raes 2012). Competitive interactions and the production of antimicrobial compounds play an important role in controlling pathogen density and disease dynamics (Wei et al. 2015). Further experimentation is needed to decipher the impact of competitive microbes on soil microbial ecological networks and plant health.

The greater variety of potential key taxa observed in the rhizosphere samples might be beneficial to maintain plant health. Analyses of LEfSe and network analysis showed that *Flavobacterium* and *Pseudomonas* may be the most active microbial species in healthy soil. *Flavobacterium* can play a role in biological control by producing antibacterial effect factors, antibacterial substances, extracellular macromolecular degrading enzymes, etc. (Bernardet and Nakagawa 2006; Kwak et al. 2018; Carrion et al*.* 2019). *Pseudomonas* which can produce antifungal/inhibitory compounds and siderophores that can control against bacterial wilt disease (Ramesh and Phadke 2012; Chandrasekaran et al. 2016). High *Pseudomonas* diversity can reduce *R. solanacearum* density in the rhizosphere and decrease the disease incidence due to both intensified resource competition and interference with the pathogen (Hu et al. 2016). Notably, the network analysis revealed the *Mortierella* and *Fusarium* were key species in healthy soils. It was also reported from previous studies that *Mortierella* was an indicator species in disease suppressive soils (Expósito et al. 2017; Xiong et al. 2017). *Mortierella* can produce antibiotics, and has potential antagonist activity against various plant pathogens (Tagawa et al. 2010). *F. oxysporum* confers biocontrol against root diseases in various plants (Lamo and Takken 2020). Thus, a potentially beneficial microbiome may form cooperative associations with other taxa to maintain plant health.

In conclusion, our results showed that there are significant differences in microbial composition between healthy and diseased soils. Both factors of soil conditions and tobacco growth periods can have an influence on the bulk and rhizosphere microbial composition. Yet, the impact of soil conditions is larger than that of tobacco growth periods in the rhizosphere soils. Discriminative taxa determined by LEfSe and network analysis in healthy soils showed beneficial potentials. This implies that steering soil microbiome in a beneficial way could have great opportunities to maintaining soil-borne disease. However, these findings need to be further confirmed in greenhouse experiments.

## Supplementary Information

**Additional file 1: Fig. S1**. PCoA of the rhizosphere soil a) bacterial and b) fungal community compositions. Abbreviations as in Fig. 2. **Table S1**. Geographic information of sampling sites. **Table S2**. Alpha-diversity indices of soil bacterial and fungal community (mean ± SE, N = 3). **Table S3**. Results of PERMANOVA analysis showing effects of different factors on soil1 microbial composition. **Table S4**. Results of PERMANOVA analysis showing effects of different factors on rhizosphere2 microbial composition. **Table S5**. Co-occurrence network properties of rhizosphere microbial communities in different samples. (DOCX 91 KB)

## Data Availability

All data generated or analyzed during this study are included in this published article and its additional files. Sequence datasets for all the samples were deposited into the NCBI short-reads archive database under accession number SRP270966.

## Abbreviations

HM/DM: Soils collected from healthy (H) and diseased (D) fields in March
HJ/DJ: Soils collected from healthy (H) and diseased (D) fields in July
HS/DS: Soils collected from healthy (H) and diseased (D) fields in September
OTU: Operational taxonomic units
PCoA: Principal coordinate analysis
PERMANOVA: Permutational multivariate analyses of variance
LEfSe: Linear discriminate analysis effect size
LDA: Linear discriminant analysis
