# Supplementary material for: Soil conditions on bacterial wilt disease affect bacterial and fungal assemblage in the rhizosphere
Source: AMB Express. 2022 Aug 29;12:110. doi: 10.1186/s13568-022-01455-1 (PMC9424452; doi:10.1186/s13568-022-01455-1)
Supplement: Supplementary file 1 — Additional file 1: Fig. S1. PCoA of the rhizosphere soil a) bacterial and b) fungal community compositions. Abbreviations as in Fig. 2. Table S1. Geographic information of sampling sites. Table S2. Alpha-diversity indices of soil bacterial and fungal community (mean ± SE, N = 3). Table S3. Results of PERMANOVA analysis showing effects of different factors on soil1 microbial composition. Table S4. Results of PERMANOVA analysis showing effects of different factors on rhizosphere2 microbial composition. Table S5. Co-occurrence network properties of rhizosphere microbial communities in different samples. (DOCX 91 KB) [file 13568_2022_1455_MOESM1_ESM.docx]

**Supplementary information**


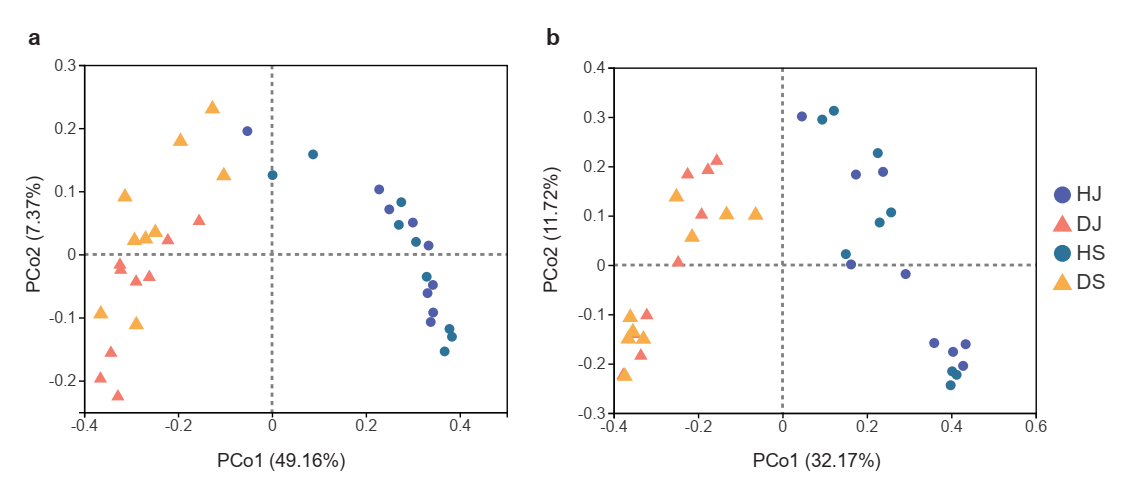


**Fig. S1** PCoA of the rhizosphere soil a) bacterial and b) fungal community compositions. Abbreviations as in Fig. 2.

**Table S1** Geographic information of sampling sites.

| Fields | Site ID | Latitude  (N) | Longitude  (E) | Altitude  (m) | Average annual temperature (°C) | Average annual precipitation (mm) |
| --- | --- | --- | --- | --- | --- | --- |
| Healthy | Site Q1 | 31°10′ | 110°02′ | 1049 | 13.7 | 1132 |
|  | Site Q2 | 31°11′ | 110°03′ | 1200 |  |  |
|  | Site Q3 | 31°13′ | 110°02′ | 1315 |  |  |
| Diseased | Site W1 | 29°08′ | 107°56′ | 1207 | 15.4 | 1104 |
|  | Site W2 | 29°15′ | 108°42′ | 1095 |  |  |
|  | Site W3 | 29°23′ | 107°24′ | 1106 |  |  |

**Table S2** Alpha-diversity indices of soil bacterial and fungal community (mean ± SE, N = 3).

|  | Collection time | Fields | Sobs index | Shannon index | Ace index | Chao1 index |
| --- | --- | --- | --- | --- | --- | --- |
| Bacteria | |  |  |  |  |  |
|  | March | Healthy | 2945.8±176.2 | 6.53±0.17 | 4400.3±291.0 | 4256.6±237.8 |
|  |  | Diseased | 3108.8±320.2 | 6.61±0.22 | 4791.4±496.9 | 4504.1±396.0 |
|  | July | Healthy | 3022.3±124.2 | 6.81±0.08* | 4153.6±245.7 | 4096.7±249.1 |
|  |  | Diseased | 2720.9±415.5 | 6.41±0.37 | 3864.5±610.1 | 3867.3±593.8 |
|  | September | Healthy | 3056.1±191.0 | 6.74±0.17 | 4251.9±294.8 | 4239.0±269.6 |
|  |  | Diseased | 3078.3±313.1 | 6.74±0.21 | 4423.0±277.2 | 4350.4±375.7 |
| Fungi | |  |  |  |  |  |
|  | March | Healthy | 366.2±142.5 | 2.56±1.03 | 663.0±221.9* | 540.6±191.8* |
|  |  | Diseased | 592.9±132.2 | 3.32±1.24 | 868.7±113.0 | 813.7±137.5 |
|  | July | Healthy | 495.6±54.5 | 3.51±0.24* | 898.2±91.8 | 749.7±77.1 |
|  |  | Diseased | 433.3±101.6 | 2.75±0.47 | 776.8±211.3 | 661.8±164.8 |
|  | September | Healthy | 481.3±47.8* | 3.30±0.28* | 785.8±95.6 | 688.7±72.81 |
|  |  | Diseased | 366.2±142.5 | 2.56±1.03 | 663.0±221.9* | 540.6±191.8* |

Note: asterisks indicated significance (P < 0.05) between healthy and diseased soils in each collection time according to independent t-test. Abbreviations as in Figure 1.

**Table S3** Results of PERMANOVA analysis showing effects of different factors on soil^1^ microbial composition.

| Region | Factor | Sum of Squares | Mean Squares | F statistic | R^2^ | P-value |
| --- | --- | --- | --- | --- | --- | --- |
| 16S | Soil conditions | 1.80 | 1.80 | 14.04 | 0.21 | 0.001 |
|  | Tobacco growth periods | 1.07 | 0.53 | 3.69 | 0.13 | 0.002 |
| ITS | Soil conditions | 2.70 | 2.70 | 10.43 | 0.17 | 0.001 |
|  | Tobacco growth periods | 2.21 | 1.10 | 4.03 | 0.14 | 0.001 |

Note: soil^1^ here indicated the soil samples collected in March, July and September.

**Table S4** Results of PERMANOVA analysis showing effects of different factors on rhizosphere^2^ microbial composition.

| Region | Factor | Sum of Squares | Mean Squares | F statistic | R^2^ | P-value |
| --- | --- | --- | --- | --- | --- | --- |
| 16S | Soil conditions | 2.60 | 2.60 | 26.01 | 0.43 | 0.001 |
|  | Tobacco growth periods | 0.14 | 0.14 | 0.80 | 0.02 | 0.473 |
| ITS | Soil conditions | 2.69 | 2.69 | 13.83 | 0.29 | 0.001 |
|  | Tobacco growth periods | 0.17 | 0.17 | 0.62 | 0.02 | 0.788 |

Note: rhizosphere^2^ here indicated the soil samples collected in July and September.

**Table S5** Co-occurrence network properties of rhizosphere microbial communities in different samples.

|  | Network indexes | July | | September | |
| --- | --- | --- | --- | --- | --- |
|  |  | Healthy | Diseased | Healthy | Diseased |
| Bacteria | Total nodes | 119 | 79 | 112 | 77 |
|  | Total links | 226 | 125 | 207 | 91 |
|  | R square of power-law | 0.89 | 0.69 | 0.87 | 0.77 |
|  | Average degree | 3.80 | 3.17 | 3.70 | 2.36 |
|  | Average clustering coefficient | 0.11 | 0.05 | 0.07 | 0.05 |
|  | Average path distance | 3.41 | 3.90 | 3.69 | 5.03 |
|  | Centralization of betweenness | 0.10 | 0.16 | 0.12 | 0.16 |
|  | Centralization of degree | 0.09 | 0.08 | 0.10 | 0.06 |
|  | Maximal stress centrality | 2371 | 1282 | 3039 | 796 |
|  | Connectedness | 0.89 | 1.00 | 0.93 | 0.83 |
|  | Positive edges | 57.78% | 58.87% | 58.74% | 60% |
|  | Negative edges | 42.22% | 41.13% | 41.26% | 40% |
| Fungi | Total nodes | 70 | 63 | 81 | 65 |
|  | Total links | 125 | 115 | 163 | 128 |
|  | R square of power-law | 0.68 | 0.45 | 0.61 | 0.28 |
|  | Average degree | 3.57 | 3.65 | 4.03 | 3.94 |
|  | Average clustering coefficient | 0.11 | 0.19 | 0.19 | 0.17 |
|  | Average path distance | 3.74 | 3.79 | 3.60 | 3.40 |
|  | Centralization of betweenness | 0.17 | 0.12 | 0.27 | 0.21 |
|  | Centralization of degree | 0.16 | 0.07 | 0.17 | 0.08 |
|  | Maximal stress centrality | 1509 | 1515 | 2634 | 1767 |
|  | Connectedness | 1.00 | 1.00 | 1.00 | 0.91 |
|  | Positive edges | 62.1% | 61.4% | 62.35% | 66.14% |
|  | Negative edges | 37.9% | 38.6% | 37.65% | 33.86% |

Note: connections were drawn between nodes that were significantly (P < 0.01; Spearman's rank correlation test) and highly (Spearman's r > 0.95) correlated.
